# Clinical Outcomes and Selection Criteria for Prodromal Huntington's Disease Trials

**DOI:** 10.1002/mds.28222

**Published:** 2020-07-20

**Authors:** Douglas R. Langbehn, Steven Hersch

**Affiliations:** ^1^ Department of Psychiatry University of Iowa Carver College of Medicine Iowa City Iowa USA; ^2^ Voyager Therapeutics, Inc. Cambridge Massachusetts USA; ^3^ Department of Neurology Massachusetts General Hospital Boston Massachusetts USA

**Keywords:** clinical trial design, premanifest Huntington's disease, prognostic index

## Abstract

**Background:**

Huntington's disease (HD) develops in individuals with extended cytosine‐adenine‐guanine (CAG) repeats within the huntingtin (*HTT*) gene, causing neurodegeneration and progressive motor and cognitive symptoms. The inclusion of mutant *HTT* carriers in whom overt symptoms are not yet fully manifest in therapeutic trials would enable the development of treatments that delay or halt the accumulation of significant disability.

**Objectives:**

The present analyses assess whether screening prediagnosis (preHD) individuals based on a normalized prognostic index (PIN) score would enable the selection of prodromal preHD subjects in whom longitudinal changes in established outcome measures might provide robust signals. It also compares the relative statistical effect size of longitudinal change for these measures.

**Methods:**

Individual participant data from 2 studies were used to develop mixed effect linear models to assess longitudinal changes in clinical metrics for participants with preHD and PIN‐stratified subcohorts. Relative effect sizes were calculated in 5 preHD studies and internally normalized to evaluate the strength and consistency of each metric across cohorts.

**Results:**

Longitudinal modeling data demonstrate the amplification of effect sizes when preHD subcohorts were selected by PIN score thresholds of >0.0 and >0.4. These models and relative effect sizes across 5 studies consistently indicate that the Unified Huntington's Disease Rating Scale total motor score exhibits the greatest change in preHD.

**Conclusions:**

These analyses suggest that the employment of PIN scores to homogenize and stratify preHD cohorts could improve the efficiency of current outcome measures, the most robust of which is the total motor score. © 2020 The Authors. *Movement Disorders* published by Wiley Periodicals LLC on behalf of International Parkinson and Movement Disorder Society

Current therapeutic trials in Huntington's disease (HD) primarily target patients with clinically diagnosed illness and mild to moderate impairment. However, a major goal for interventional therapy in HD is preventive treatment prior to the onset of disabling symptoms.^1^ Expanding clinical trial design to enable inclusion of prediagnosis cohorts with observable disease features could help achieve this goal.

In HD, the development of clinical signs and symptoms is insidious, accumulating until a clinician is completely certain that a diagnosis is warranted. Individuals carrying mutant *HTT* who have not yet received a clinical diagnosis encompass the preHD population. A subset of preHD is the prodromal HD population, which includes individuals with subtle symptomology that does not yet support diagnostic certainty. Studies such as TRACK‐HD,^2^ Neurobiological Predictors of Huntington's Disease (PREDICT‐HD),^3^ and Prospective Huntington at Risk Observational Study (PHAROS)^4^ clearly demonstrate the subtle decline of cognitive and motor function years in advance of overt clinical illness, along with a concomitant loss of brain volume both prior to diagnosis and throughout disease progression.^5^, ^6^ Indeed, smaller basal ganglia volumes are present in preHD individuals long before the predicted time of symptom manifestation (although developmental effects could account for some of the volumetric differences).^7^, ^8^ The significant clinical impact of prodromal HD is also becoming better understood.^9^

The imperative for more efficient clinical trial design is accentuated by the ongoing development of compelling experimental HD treatments that could modify its course, including gene‐targeted therapies to decrease the translation of mutant huntingtin protein,^10^ stem‐cell replacement of damaged or vulnerable brain tissue,^11^ as well as biological or small‐molecule drugs targeting pathophysiologic mechanisms.^12^ The patient populations for most therapeutic trials have usually been stage I and II (early stage, clinically diagnosed) as defined by the total functional capacity (TFC) score,^13^ which is a subscale of the Unified Huntington's Disease Rating Scale (UHDRS).^14^ The choice has primarily been based on the anticipated benefit/risk profile in a patient population for whom disease progression is in its early stages, quality of life remains relatively high, and symptom‐based outcome measures are amenable to demonstrating a clinically meaningful treatment effect. However, many years of neurodegenerative changes precede the clinical diagnosis of HD, and disease‐modifying treatments could delay or prevent the development of early disability if applied during the preHD period.

The insidious initial decline in preHD presents a major challenge for trial design. If a trial were to include a random sample of all preHD^15^ individuals, vastly more study participants and/or longer observation periods would be required than are typically needed for efficacy trials in patients with stage I or II disease.^16^ Successful trials in preHD depend on identifying a combination of valid outcome measures and a preHD subgroup that, in the absence of treatment, will have observable progression in a trial of feasible size and duration. In this article, we focus on both elements of this requirement: the choice of outcome and the criteria for enriching enrollment for those likely to show measurable change on that outcome.

To be useful, a primary outcome measure must reliably detect longitudinal change among untreated individuals. Clinical relevance is also essential, but impossible if change is not detectable. The focus of this report is detectability of change. Here we assess the motor and cognitive measures from the UHDRS as outcome measure candidates for preHD populations.^14^ We examined these measures in 5 preHD studies that used the UHDRS. We also examined additional pen‐and‐paper cognitive measures administered in some of these studies. Finally, we calculated the preHD progression of a recently proposed composite score of UHDRS items (cUHDRS) that is already in use in clinical trials of early diagnosed HD.^17^, ^18^

To address enrichment, we begin with the knowledge that the measures under consideration change more rapidly further into the prodromal period. Historically, level of prodromal risk has been measured by genetic burden score or the estimated years until clinically significant onset. Both measures are based on the length of the cytosine‐adenine‐guanine (CAG) trinucleotide expansion in the *HTT* gene and the person's age. Long and colleagues^18^ recently showed that onset prediction is further enhanced by a risk score that incorporates the total motor score (TMS) and symbol digit modalities test (SDMT) from the UHDRS as well as CAG length and age. The resulting normalized prognostic index (PIN) score has proven an effective predictor of time until HD diagnosis when applied retrospectively in a number of studies.^18^, ^19^ Here, we report the increase in predictable candidate outcome changes observed using 2 different screening PIN thresholds for enriching trial entry criteria.

## Methods

### Data Sources

Retrospective analyses were performed on data cuts from 2 studies and included a total of 3974 preHD participants. ENROLL‐HD (NCT01574053) is a global clinical research platform designed to facilitate clinical research in HD. Core data sets are collected annually from all research participants as part of this multicenter, longitudinal observational study. Data are monitored for quality and accuracy using a risk‐based monitoring approach. All sites are required to obtain and maintain local ethical approval. ENROLL‐HD includes centers across Europe, Australasia, and the Americas that combine populations from the REGISTRY and Cooperative Huntington's Observational Research Trial (COHORT) studies with additional sites. Data from 3557 preHD participants (mean age = 39.8; mean CAG repeat length = 42.4) who were enrolled between July 2012 and October 2018 were included in the present analysis.^20^ Raw individual participant data for the ENROLL‐HD December 2018 data cut were available and enabled primary analyses of outcome metrics stratified by PIN scores. The COHORT study (NCT00313495) enrolled participants in the United States, Canada, and Australia between 2006 and 2011. The mean age among 417 participants with preHD was 41.1, and their mean CAG repeat length was 42.4.^21^ Raw individual participant data for the COHORT December 31, 2009 data cut were available and enabled primary analyses of outcome metrics stratified by PIN scores.

Additional secondary analyses were based on published results from 3 other studies. TRACK‐HD was a longitudinal observational study that included multiple sites across Europe and Canada that began enrolling in January 2008 and ended in September 2011. Data extracted for the present analysis were from 120 preHD participants (mean age = 41.0; mean CAG repeat length = 43.0).^2^, ^22^ Published effect sizes were used to calculate effect sizes presented in this analysis.^2^ The PREDICT‐HD (NCT00051324) study enrolled 1013 preHD participants between 2001 and 2012 across 32 sites, among whom the mean age was 41.0 and the mean CAG repeat length was 42.3.^23^ Relative effect sizes were derived from the likelihood ratio test statistics for variables tabulated by Paulsen and colleagues.^23^ PHAROS (NCT00052143) was a longitudinal cohort study that collected data from July 9, 1999, to December 17, 2009. The 345 preHD enrollees with a CAG expansion (CAG repeat length ≥ 37) had a mean age of 42.2 years.^4^ The 3‐year effect sizes from the 2015 report of Biglan and colleagues^4^ were used to calculate the relative effect sizes presented here.

From these 3 latter studies, we extracted the following measures, all used in ENROLL‐HD, when available: UHDRS TMS, UHDRS TFC, SDMT, Stroop tests (word condition, color condition, and interference condition), Trail‐Making Tests Parts A and B, and verbal fluency tests (letter and category). These cognitive variables were limited to those also used in ENROLL‐HD. Effect sizes of additional cognitive variables from the TRACK‐HD and PREDICT‐HD publications were all smaller than the SDMT effect sizes from those studies.

### Longitudinal Effect Size Modeling

With the data available for the ENROLL‐HD and COHORT studies, we calculated baseline visit PIN scores from the number of CAG trinucleotide repeats, age, TMS, and SDMT scores from each participant (as described by Long and colleagues^18^). To avoid potential misclassification, we excluded 408 ENROLL‐HD participants (11.5%) and 33 COHORT participants (7.3%) from the present analyses because they had baseline TMS >20 or TFC <11 despite being classified as preHD by the local clinical researcher. (Those who reach these thresholds are often considered past the prodromal preHD stage by many clinicians.) PIN score minima of 0.0 and 0.4 were applied to the remaining preHD population for additional high‐risk subpopulation analyses. We chose these thresholds because they represented the approximate top half and top tercile of risk within ENROLL‐HD (49.1% and 34.0%) and COHORT (50.5% and 36.2%). Furthermore, a PIN score of 0.0 predicted a 50% 10‐year probability of receiving a motor diagnosis within the PREDICT‐HD study.^18^ The cUHDRS score was calculated from the TMS, TFC, SDMT, and Stroop word condition assessments to evaluate its longitudinal statistical signal.^17^

Individual participant data were available for the ENROLL‐HD and COHORT studies with approximately annual follow‐up. We analyzed a maximum of 3.5 years of follow‐up data per participant to approximate progression that would be seen in a realistic clinical trial timeframe. Mixed effect linear models with correlated random intercepts and slopes were applied to estimate the adjusted mean slopes of longitudinal changes in clinical metrics. We also obtained the estimated covariance of random intercepts and slopes from these models. We translated these statistics to effect sizes over a hypothetical 3‐year trial with follow‐up every 3 months. Effect size is the estimated mean change over 3 years divided by the standard deviation of participants’ individual estimated mean rates of change. This time‐dependent standard deviation is derived from the estimated covariance of random effects from the mixed effect model. *P* values attached to effect sizes are the mixed effect model *P* values for the slopes of outcome change and are approximate.

We define a hypothetical treatment effect as the proportion by which longitudinal decline is assumed to slow. We translated the previous effect sizes to corresponding approximate sample sizes required for a 2‐armed clinical trial, assuming a given treatment effect and follow‐up every 3 months.^24^ These calculations ignore details such as expected drop‐out rates or the alternative possibilities of unbalanced or adaptive designs. However, we believe they are first‐order approximations sufficient to illustrate the order of magnitude for a realistic number of trial participants and to demonstrate the relevant efficiencies of various candidate outcomes.

For other published trials, we derived longitudinal effect sizes relative to TMS effect size, as sufficient detail was not available to calculate the absolute effect sizes, and the cUHDRS scores could not be calculated. For TRACK‐HD and PHAROS, the relative effect sizes reported are the ratios of the corresponding published *t* statistics for the significance of longitudinal change. For PREDICT, only likelihood statistics and nonmissing sample sizes were available. Effect size estimates reported are the square roots of likelihood‐per‐subject ratios.

## Results

**FIG. 1:**
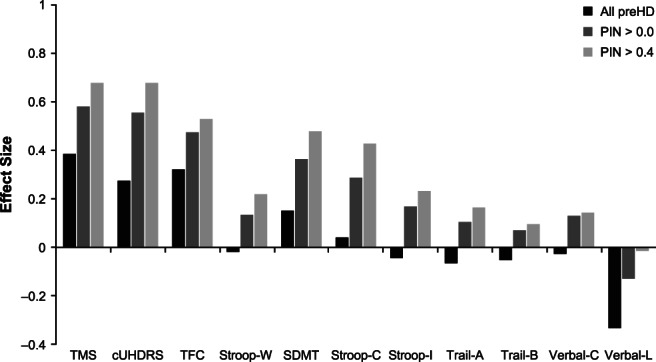
Three‐year effect sizes of assessment metrics in preHD populations from ENROLL‐HD. cUHDRS, composite Unified Huntington's Disease Rating Scale; PIN, prognostic index; preHD, prediagnosis Huntington's disease; SDMT, Symbol Digit Modalities Test; Stroop‐C, Stroop Color and Word Test (color condition); Stroop‐I, Stroop Color and Word Test (interference condition); Stroop‐W, Stroop Color and Word Test (word condition); TFC, total functional capacity; TMS, total motor score; Trail‐A, trail making test part A; Trail‐B, trail making test part B; Verbal‐C, categorical verbal fluency test; Verbal‐L, letter verbal fluency test.

**FIG. 2:**
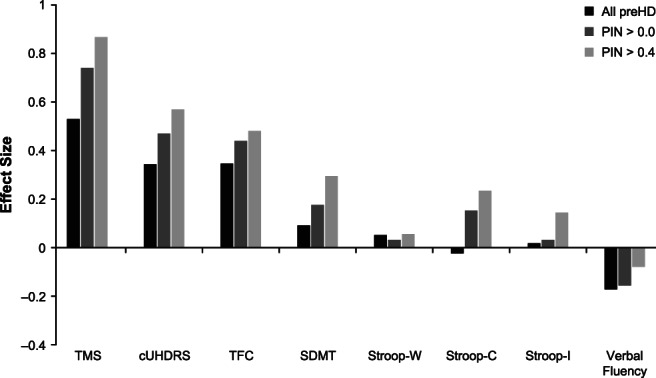
Three‐year effect sizes of assessment metrics in preHD populations from the Cooperative Huntington's Observational Research Trial. cUHDRS, composite Unified Huntington's Disease Rating Scale; PIN, prognostic index; preHD, prediagnosis Huntington's disease; SDMT, Symbol Digit Modalities Test; Stroop‐C, Stroop Color and Word Test (color condition); Stroop‐I, Stroop Color and Word Test (interference condition); Stroop‐W, Stroop Color and Word Test (word condition); TFC, total functional capacity; TMS, total motor score.

In both the ENROLL‐HD (Fig. 1) and COHORT (Fig. 2) total preHD populations (all preHD), the effect sizes were consistently larger for clinical measures incorporating motor functions (cUHDRS and TMS) compared with TFC or any of the available cognitive measures (verbal fluency, trail making, Stroop Word and Color, and SDMT tests). TMS on its own had the largest effect size over a 3‐year timespan in each study.

Reanalyzing subpopulations based on threshold PIN scores (PIN >0.0 and PIN >0.4), effect sizes were amplified in the higher risk groups for most outcome measures (Figs. 1 and 2). The effect sizes for the most sensitive (3‐year effect size >0.2) metrics of TFC, cUHDRS, and TMS all increased substantially with higher PIN thresholds. Some cognitive measures also exhibited more detectable changes among subpopulations restricted to participants with higher PIN scores. It is notable that SDMT and Stroop color condition tests achieved effect sizes >0.2 in higher risk groups from both cohorts, whereas ENROLL‐HD data also support the higher sensitivity of Stroop word condition and Stroop interference condition tests in subpopulations defined by their PIN scores.

**FIG. 3:**
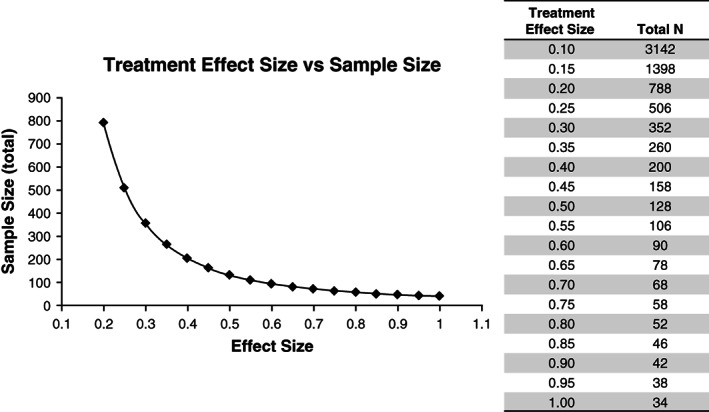
Required sample sizes for 2‐group comparisons for given treatment effect sizes.^a^ The figure contains sample sizes for a *t* test, 80% power, and a significance level (*P* value) = .05. Total N is the combined sample size for 2 groups, assuming 50% of participants per group. ^a^These sample sizes should be considered rough generic estimates. A realistic trial size determination would consider additional effects such as assumed drop‐out rates, possible uneven treatment–placebo allotment ratios, and placebo effects. Crucially, a realistic trial size also depends on defining a suitable clinically meaningful treatment effect to be detected. The example of 50% is meant only for illustration of sample size principles.

The approximate translation of treatment effect sizes to sample sizes is illustrated in Figure 3, assuming a *P* value of 0.05 and 80% power. To illustrate, if we take as an example an effect size of natural decline of 0.80 SD over the duration of the study and a target treatment effect of 50% slowing, then sample size would be calculated by the following formula: Treatment effect size = (0.80 * 0.50) = 0.40. The trial would therefore require roughly 200 participants. Supplementary Tables 1 and 2 list the natural decline effect sizes illustrated in Figures 1 and 2 and their translation to approximate sample sizes required to detect a treatment effect of 50% slowing of decline.

Based on published data, we calculated the relative preHD effect sizes (Table 1) and required study‐enrollment numbers (Table 2) compared to TMS for other measures reported in the TRACK‐HD, PREDICT‐HD, and PHAROS studies. Consistent with the ENROLL‐HD and COHORT analyses, the TMS retained the strongest signal in each of the other studies.

**TABLE 1:** Relative effect sizes among prediagnosis Huntington's disease participants from 5 studies

Outcome	ENROLL‐HD	COHORT	TRACK‐HD^*^	PREDICT‐HD^**^	PHAROS^***^
TMS	1.000	1.000	1.000	1.000	1.000
TFC	0.836	0.655	0.090	0.546	0.216
SDMT	0.392	0.176	0.504	0.881	0.661
Stroop‐W	−0.050	0.105	0.425	0.756	–
Stroop‐C	0.110	−0.043	–	0.775	–
Stroop‐I	−0.110	0.037	–	0.665	–
Verbal‐C	−0.068	–	–	–	–
Verbal‐L	−0.872	−0.320	–	–	–
Trail‐A	0.164	–	–	0.604	–
Trail‐B	0.133	–	–	0.678	–

–, not assessed.

COHORT, Cooperative Huntington's Observational Research Trial; PREDICT**‐**HD, Neurobiological Predictors of Huntington's Disease; PHAROS, Prospective Huntington at Risk Observational Study; TMS, total motor score; TFC, total functional capacity; SDMT, Symbol Digit Modalities Test; Stroop‐W, Stroop Color and Word Test (word condition); Stroop‐C, Stroop Color and Word Test (color condition); Stroop‐I, Stroop Color and Word Test (interference condition); Verbal‐C, categorical verbal fluency test; Verbal‐L, letter verbal fluency test; Trail‐A, Trail Making Test Part A; Trail‐B, Trail Making Test Part B.

^*^ Three‐year effect sizes relative to controls for nearer to onset preHD (preHD B) published in Tabrizi et al.^2^

^**^ Approximate relative effect sizes relative to controls derived from likelihood ratio statistics in Table 2 of Paulsen et al.^3^

^***^ Three‐year effect sizes relative to controls calculated from Table 3 in Huntington Study Group, PHAROS Investigators.^4^

**TABLE 2:** Estimated required sample sizes relative to using TMS as the outcome in a 3‐year trial

Outcome	ENROLL‐HD	COHORT	TRACK‐HD	PREDICT‐HD	PHAROS
TMS	1.00	1.00	1.00	1.00	1.00
TFC	1.43	2.33	123	3.35	21.43
SDMT	6.51	32.3	3.94	1.29	2.29
Stroop‐W	a	90.7	5.54	1.75	–
Stroop‐C	82.6	a	–	1.66	–
Stroop‐I	a	730	–	2.26	–
Verbal‐C	a	–	–	–	–
Verbal‐L	a	a	–	–	–
Trail‐A	37.2	–	–	2.74	–
Trail‐B	56.5	–	–	2.18	–

–, not assessed in Table 1.

^a^ Negative effect sizes in Table 1, therefore no sample size estimable.

COHORT, Cooperative Huntington's Observational Research Trial; PREDICT**‐**HD, Neurobiological Predictors of Huntington's Disease; PHAROS, Prospective Huntington at Risk Observational Study; TMS, total motor score; TFC, total functional capacity; SDMT, Symbol Digit Modalities Test; Stroop‐W, Stroop Color and Word Test (word condition); Stroop‐C, Stroop Color and Word Test (color condition); Stroop‐I, Stroop Color and Word Test (interference condition); Verbal‐C, categorical verbal fluency test; Verbal‐L, letter verbal fluency test; Trail‐A, Trail Making Test Part A; Trail‐B, Trail Making Test Part B.

## Discussion

The efficient assessment of potential disease‐modifying therapies in the preHD population would be greatly enhanced by predictive measures of disease progression and validation of primary outcome measures appropriate for this population. In our analyses, the use of PIN score thresholds effectively identified the participants most likely to show short‐term (≤3 years) progression on select outcomes. This demonstrates the potential of PIN thresholds to enrich trial populations to include participants with more homogeneous, detectable disease progression. The results, consistent among 5 large studies of preHD, also showed that an increase in the UHDRS TMS is the most readily measured change in preHD among the commonly used clinical HD assessments, regardless of whether enrichment strategies are used. Our selection of a conservative filter in our analyses of ENROLL‐HD and COHORT to exclude potential early, misclassified manifest HD participants further supports this conclusion.

The strength of the TMS longitudinal signal suggests that sample sizes and study durations for clinical trials in the prodromal HD population could be minimized by using the TMS as the primary outcome measure. Although the TMS is primarily a measure of motor signs, its progression in preHD may be representative of overall prodromal progression because motor, cognitive, and neuroimaging outcomes are all known to progress through premanifest and early manifest HD. According to recent analyses of TRACK‐HD data, the progression of both motor and cognitive measures correlates strongly with brain volume loss.^25^ The TMS may simply be the most sensitive clinical measure of prodromal HD and could thus serve as a proxy for overall early disease progression. Nevertheless, it would be important to use clinical trial designs that can help distinguish symptomatic from disease‐modifying effects on the TMS.^26^ Longer trial durations that can assess whether treatment effects outlive short‐term symptomatic responses, assess the persistence of TMS scores after treatment withdrawal, and control for concomitant medications that can modulate motor symptoms would all be considerations to ensure the results distinguish symptomatic from disease‐modifying effects. Slowing progressive regional brain atrophy or other biomarkers in concert with TMS progression could provide additional evidence of effectiveness. For example, basal ganglia volume change is consistently measurable in individuals predicted to be decades away from clinical illness.^8^ However, the effect of PIN score stratification upon the relationships between neuroimaging outcomes and prodromal TMS progression have not yet been studied.

Collections of multidomain metrics, such as the cUHDRS,^17^ have been developed to assess progression in manifest disease with heightened robustness and broader clinical representativeness. However, in preHD, neither ENROLL‐HD nor COHORT demonstrated an improved longitudinal statistical signal with the cUHDRS over the TMS alone. Effect sizes were similar in the ENROLL‐HD data, but the cUHDRS had a notably weaker effect than TMS in the COHORT data. Although its statistical signal was not stronger, the clinical validity of the cUHDRS may be broader because it incorporates cognitive and functional domains as well as motor. This would be supported if progression of cUHDRS signal in preHD reflected substantial changes in each of its parts. However, the available data are inconsistent regarding which of the cUHDRS measures contribute to a preHD signal (Tables S1 and S2).^20^, ^21^ ENROLL‐HD data show TFC, TMS, and to a lesser extent SDMT changes contributing; in COHORT, the additional signal is primarily attributed to the TFC. Among the other historical data sets, all 3 additional measures showed notable change in PREDICT‐HD, whereas there were minimal contributions from the TFC in TRACK‐HD and PHAROS.

The TFC has inherent face validity as an outcome; however, the functional domains evaluated would not typically decline prior to clinical illness. Therefore, the contribution of the TFC to the cUHDRS might represent a proxy measure for “diagnosis” of HD. We considered whether the strength of the TFC signal among participants classified as preHD is attributed to misclassification of some participants who had unaccounted for delays in diagnosis, suggesting the contribution would disappear in a stringently defined preHD cohort. We limited this in our ENROLL‐HD and COHORT analyses by excluding participants who had baseline TMS >20 or TFC scores <11, despite not being diagnosed; still, we found appreciable TFC signal in those studies. In contrast, the estimated TFC contribution was minimal in TRACK‐HD, which was quite stringent in excluding those with TMS >5 from the preHD cohort. Thus the “cleanest” available preHD data had almost no change in TFC performance, as intuitively expected. TFC may only be a valuable measure in the intermediate cohort showing some appreciable HD signs ahead of diagnostic certainty (ie, a preHD group with high PIN scores). It follows that the TFC could contribute to change in cUHDRS scores for that group.

The SDMT and Stroop tests are primarily intended to measure cognitive function, although motor impairment affecting the response times can be confounding,^27^ at least in diagnosed HD. It is unclear if subtle motor effects may play a similarly confounding role in preHD. The SDMT also contributes to the PIN score calculation; thus, the prognostic validity of baseline SDMT scores has been established in preHD.^18^ However, the effect size associated with prospective change from those baseline scores varied widely among the studies that we analyzed (Table 1).

Regarding other cognitive tests, none that we examined in ENROLL‐HD or COHORT showed substantial effect sizes in preHD. In several instances these tests registered a negative effect, which means that the scores improved longitudinally. This is consistent with the practice effect improvement routinely seen in control volunteers, even with tests administered only once per year. Small but statistically significant differences in practice effects between preHD and controls have been measured, and longitudinal effect sizes are sometimes calculated relative to improving performance in controls, as in TRACK‐HD, PREDICT‐HD, and PHAROS (Table 1). Cognitive outcome effect sizes in those studies are notably larger than in ENROLL‐HD or COHORT but are still smaller than the accompanying TMS effect sizes. Practice effects may not represent the same aspect of cognition that the test is intended to measure, complicating interpretation of a therapy's effects. The disparate estimates of cognitive test effect size in Tables 1 and 2 should be considered best‐case and worst‐case scenarios, depending on whether a treatment also improved practice effects.

Our results may be limited by the candidate outcomes available for our analyses as well as the nature of the data included. In contrast to the observational data examined here, results of analyses from controlled clinical trials could differ as a result of placebo effects, minimization of practice effects by pretrial repetition, and the differing motivations of both participants and raters. The applicability of data from trials that have included patients diagnosed with HD may also be limited because the magnitudes of impairment and rates of change are much larger than would be anticipated in preHD. For example, in a recent pridopidine trial of patients with manifest HD, a mean placebo effect of about 5 points of TMS improvement was seen at 26 weeks.^28^ However, this occurred in a group with obvious motor impairment and TMS scores several times higher than would be encountered in preHD. In that study, quantitative motor measurements without apparent placebo effects were favorable. Nevertheless, the effect sizes of similar quantitative motor tests in the TRACK‐HD study were much smaller than the TMS signal in preHD subjects.^2^ Our current conclusions will eventually be refined as clinical trial experience with preHD individuals grow.

In conclusion, we found that the TMS had the largest longitudinal effect size among outcome measures that have been assessed in preHD in large observational studies. Although cognitive alterations may be detectable prior to motor symptoms,^23^, ^29^, ^30^ our comparisons among candidate cognitive, motor, and functional outcomes across 5 studies of preHD consistently demonstrate that the largest longitudinal effect was measured for the TMS. Furthermore, we have demonstrated that preHD PIN scores could be used to enrich participant selection for those most likely to experience measurable decline, a prerequisite for detecting a treatment effect in the rate of decline. Such enrichment substantially reduces required sample sizes for clinical trials enrolling preHD individuals.

## Author Roles

(1) Research project: A. Conception, B. Organization, C. Execution; (2) Statistical Analysis: A. Design, B. Execution, C. Review and Critique; (3) Manuscript: A. Writing of the first draft, B. Review and Critique.

D.R.L.: 1A, 1B, 1C, 2A, 2B, 3A, 3B

S.H.: 1A, 1B, 2C, 3B

## Full financial disclosures for the previous 12 months

Dr. Langbehn receives research funding from the National Institute of Neurological Disorders and Stroke, the CHDI Foundation, University College of London, and from the Wellcome Trust. He also serves or has served as a paid statistical consultant for the design of Huntington's Disease trials for F. Hoffmann‐La Roche Ltd, Voyager Therapeutics, Teva Pharmaceuticals USA, Inc., Novartis AG, Takeda Pharmaceutical Company Limited, Asklepios BioPharmaceutical, Inc. (AskBio), Wave Life Sciences and uniQure N.V. He has also been a paid consultant to Axon Advisors, LLC.

Dr. Hersch is a full‐time employee of Voyager Therapeutics and a part‐time employee of Massachusetts General Hospital.

## Supporting information


**Appendix** S1: Supporting Information.
