# Supplementary material for: Clinical Outcomes and Selection Criteria for Prodromal Huntington's Disease Trials
Source: Mov Disord. 2020 Jul 20;35(12):2193–200. doi: 10.1002/mds.28222 (PMC7818458; doi:10.1002/mds.28222)
Supplement: Supplementary file 1 — Appendix S1: Supporting Information. [file MDS-35-2193-s001.docx]

**SUPPLEMENTAL INFORMATION**

**Table S1. ENROLL-HD Effect Sizes**

|  | **All preHD** | | |  | **PIN > 0.0** | | |  | **PIN > 0.4** | | |
| --- | --- | --- | --- | --- | --- | --- | --- | --- | --- | --- | --- |
|  | **Effect Size** | ***P*** | **N** |  | **Effect Size** | ***P*** | **N** |  | **Effect Size** | ***P*** | **N** |
| **TMS** | 0.383 | < .0001 | 856 |  | 0.579 | < .0001 | 375 |  | 0.678 | < .0001 | 273 |
| **cUHDRS** | 0.274 | < .0001 | 1673 |  | 0.554 | < .0001 | 409 |  | 0.679 | < .0001 | 272 |
| **TFC** | 0.320 | < .0001 | 1226 |  | 0.472 | < .0001 | 564 |  | 0.530 | < .0001 | 447 |
| **Stroop-W** | -0.019 | 0.452 | − |  | 0.135 | 0.001 | 6891 |  | 0.220 | < .0001 | 2595 |
| **SMTD** | 0.150 | < .0001 | 5581 |  | 0.365 | < .0001 | 943 |  | 0.477 | < .0001 | 552 |
| **Stroop-C** | 0.042 | 0.099 | 71192 |  | 0.287 | < .0001 | 1525 |  | 0.425 | < .0001 | 695 |
| **Stroop-I** | -0.042 | 0.111 | − |  | 0.170 | < .0001 | 4345 |  | 0.234 | < .0001 | 2293 |
| **Trail-A** | -0.063 | 0.021 | − |  | 0.104 | 0.016 | 11611 |  | 0.166 | 0.002 | 4557 |
| **Trail-B** | -0.051 | 0.068 | − |  | 0.072 | 0.102 | 24225 |  | 0.098 | 0.073 | 13076 |
| **Verbal-C** | -0.026 | 0.287 | − |  | 0.130 | 0.001 | 7431 |  | 0.141 | 0.002 | 6317 |
| **Verbal-L** | -0.334 | < .0001 | − |  | -0.128 | 0.003 | − |  | -0.013 | 0.812 | − |

cUHDRS, composite Unified Huntington's Disease Rating Scale; PIN, prognostic index; preHD, pre-diagnosis Huntington’s disease; SDMT, symbol digit modalities test; Stroop-C, Stroop color and word test (condition: color reading); Stroop-I, Stroop Color and Word Test (condition: interference); Stroop-W, Stroop color and word test (condition: word reading); TFC, total functional capacity; TMS, total motor score; Trail-A, trail making test part A; Trail-B, trail making test part B; Verbal-C, categorical verbal fluency test; Verbal-L, letter verbal fluency test.

*P* = p value for two-sided hypothesis test that effect size is 0. N = Estimated total participants for a two-armed, balanced randomized clinical trial with assumed treatment effect of 50% reduction of progression rate. A 3-year trial with follow-up every 3 months is assumed. Sample sizes would be nonsensical for negative effect sizes.

**Table S2. COHORT Effect Sizes**

|  | **All preHD** | | |  | **PIN > 0.0** | | |  | **PIN > 0.4** | | | |
| --- | --- | --- | --- | --- | --- | --- | --- | --- | --- | --- | --- | --- |
|  | **Effect Size** | ***P*** | **N** |  | **Effect Size** | ***P*** | **N** |  | **Effect Size** | | ***P*** | **N** |
| **TMS** | 0.534 | < .0001 | 440 |  | 0.742 | < .0001 | 228 |  | 0.871 | < .0001 | | 166 |
| **cUHDRS** | 0.343 | < .0001 | 1067 |  | 0.472 | < .0001 | 564 |  | 0.574 | < .0001 | | 381 |
| **TFC** | 0.350 | < .0001 | 1025 |  | 0.443 | < .0001 | 640 |  | 0.484 | < .0001 | | 536 |
| **SDMT** | 0.094 | 0.443 | 14213 |  | 0.180 | 0.723 | 3876 |  | 0.299 | 0.608 | | 1405 |
| **Stroop-W** | 0.056 | 0.186 | 40045 |  | 0.035 | 0.068 | 102516 |  | 0.059 | 0.012 | | 36076 |
| **Stroop-C** | -0.023 | 0.751 | − |  | 0.154 | 0.127 | 5295 |  | 0.238 | 0.045 | | 2217 |
| **Stroop-I** | 0.020 | 0.778 | 313955 |  | 0.034 | 0.719 | 108635 |  | 0.146 | 0.196 | | 5891 |
| **Verbal Fluency** | -0.171 | 0.019 | − |  | -0.153 | 0.140 | − |  | -0.077 | 0.526 | | − |

cUHDRS, composite Unified Huntington's Disease Rating Scale; PIN, prognostic index; preHD, pre-diagnosis Huntington’s disease; SDMT, symbol digit modalities test; Stroop-C, Stroop color and word test (condition: color reading); Stroop-I, Stroop Color and Word Test (condition: interference); Stroop-W, Stroop color and word test (condition: word reading); TFC, total functional capacity; TMS, total motor score.

*P* = p value for two-sided hypothesis test that effect size is 0. N = Estimated total participants for a two-armed, balanced randomized clinical trial with assumed treatment effect of 50% reduction of progression rate. A 3-year trial with follow-up every 3 months is assumed. Sample sizes would be nonsensical for negative effect sizes.
